# Low-Cost Vegetal Simulation for Ear Reconstruction (Otopoiesis) Training: A Technical Report

**DOI:** 10.7759/cureus.115392

**Published:** 2026-08-29

**Authors:** Sonia Naija, Sabrina Maamouri, Feres Zehani, Yasmine Saanaa, Atf Yacoub

**Affiliations:** 1 Oral and Maxillofacial Surgery, Principal Military Hospital of Instruction of Tunis (HMPIT), Tunis, TUN; 2 Maxillofacial Surgery, Charles Nicolle Hospital, Tunis, TUN

**Keywords:** active learning, cost-effective training, low-fidelity model, medical simulation, otopoiesis, surgical education

## Abstract

Autologous ear reconstruction (otopoiesis) involves a steep learning curve because costal cartilage is a nonrenewable biological resource with no margin for error. Consequently, early preclinical training is valuable, yet high-fidelity commercial simulators remain expensive and inaccessible in resource-limited settings. This report describes the educational feasibility and implementation of a low-cost, low-fidelity vegetal simulation model (raw potato) for early-stage otopoiesis carving training within a continuing medical education workshop. Sixteen maxillofacial surgery residents (N=16), organized into junior-senior dyads, took part in an anatomical prebriefing, 2D-to-3D template transfer, and step-by-step carving of five distinct ear components based on Firmin's technique, under continuous formative supervision by two senior surgeons. Educational impact was assessed using an anonymous 10-item knowledge questionnaire administered before and after the workshop and an anonymous post-workshop satisfaction survey. Mean knowledge scores improved from 56.0% (pretest, N=15) to 80.0% (post-test, N=8), with improvement or stability across all 10 items. All satisfaction survey respondents (100%, 9/9) agreed that the workshop improved their three-dimensional spatial understanding of auricular architecture, and 77.8% (7/9) reported a clear improvement in theoretical knowledge, consistent with the objective knowledge gain. In conclusion, low-fidelity vegetal simulation appears to be a feasible, accessible, and low-cost educational tool for introducing the conceptual and procedural foundations of early-stage otopoiesis training; however, it does not replace hands-on training with higher-fidelity or biological models before live surgery.

## Introduction

Reconstructive surgery of the auricle, commonly referred to as otopoiesis, represents one of the most demanding procedures in the field of maxillofacial and plastic reconstructive surgery [1]. The intervention requires not only extraordinary anatomical precision but also highly developed three-dimensional (3D) spatial awareness. Concretely, otopoiesis involves harvesting autologous costal cartilage, carving it into a five-component auricular framework, and implanting it beneath a temporoparietal or mastoid skin flap over one or more surgical stages. The ultimate goal of the surgeon is to recreate a complex, delicate auricular structure with detailed positive and negative reliefs using autologous biological material, specifically costal cartilage harvested from the patient [2,3].

Traditionally, surgical education has relied heavily on the Halstedian apprenticeship model of “see one, do one, teach one”, the historical framework in which trainees learn a procedure through direct observation and repetition under supervision. However, modern medical ethics dictate that complex surgical procedures should never be performed for the first time on a live patient [4]. In otopoiesis, where the harvested costal cartilage graft is a nonrenewable biological resource associated with donor-site morbidity, the margin for error during the carving phase is zero [5,6]. Consequently, simulation-based medical education has become indispensable for safeguarding patient outcomes.

While high-fidelity simulators, such as three-dimensional-printed synthetic polymers or cadaveric models, provide excellent training platforms [2,7], they remain extremely expensive, technically demanding to produce, and largely inaccessible in developing regions and resource-limited academic departments. To circumvent these constraints, the development of low-cost, low-fidelity procedural simulation models that replicate essential cognitive and motor tasks without requiring perfect replicas of human tissue is highly valuable [8]. The use of common vegetables, such as potatoes, for early practical initiation in auricular carving represents a promising, frugal pedagogical approach [9]. This report describes an educational intervention utilizing a low-cost vegetal simulation model for otopoiesis training and evaluates its educational feasibility, pedagogical implementation, and early-stage utility in developing baseline conceptual and technical understanding.

## Technical report

Study setting and participants

A practical, experiential continuing medical education workshop (“cours de collège”) was organized within a single academic training department as part of the department’s recurring thematic teaching sessions for residents of all postgraduate (PGY) years. The session involved 16 maxillofacial surgery residents (N=16), ranging from the first to the fifth postgraduate year.

As a routine educational activity without a research hypothesis, randomization, control group, or comparison of clinical outcomes, formal Institutional Review Board approval was not required in accordance with institutional policy. Participation was voluntary, and residents were informed that anonymized session data (knowledge questionnaire and satisfaction survey responses) could be used for educational reporting purposes. No directly identifying information was collected; all quantitative data were gathered through unmarked, anonymous Google Forms.

None of the participating residents had ever independently performed an otopoiesis procedure in the operating room. Residents were organized into supervised dyads, pairing a junior trainee (PGY1-PGY3) with a senior trainee (PGY4-PGY5). Notably, senior residents also had no prior hands-on exposure to the full five-component carving sequence, making this a shared learning opportunity across seniority levels rather than a purely top-down teaching exercise. Two senior maxillofacial surgeons circulated among all dyads throughout the session, providing real-time technical correction and validating the anatomical conformity of each finished component.

Simulation model and surgical technique

The training session was structured as a focused educational intervention. It commenced with a targeted 30-minute theoretical prebriefing reviewing the anatomical principles of the cartilaginous framework of the ear and the costal origin of each anatomical component according to Firmin’s bistage reconstruction technique [10]. The materials provided to each participant included calibrated raw potato half-tubers, surgical skin markers, assorted surgical scalpel blades, bone gouges of varying diameters, 4-0 double-armed sutures, and plastic cutting boards, as illustrated in Figure 1.

**Figure 1:**
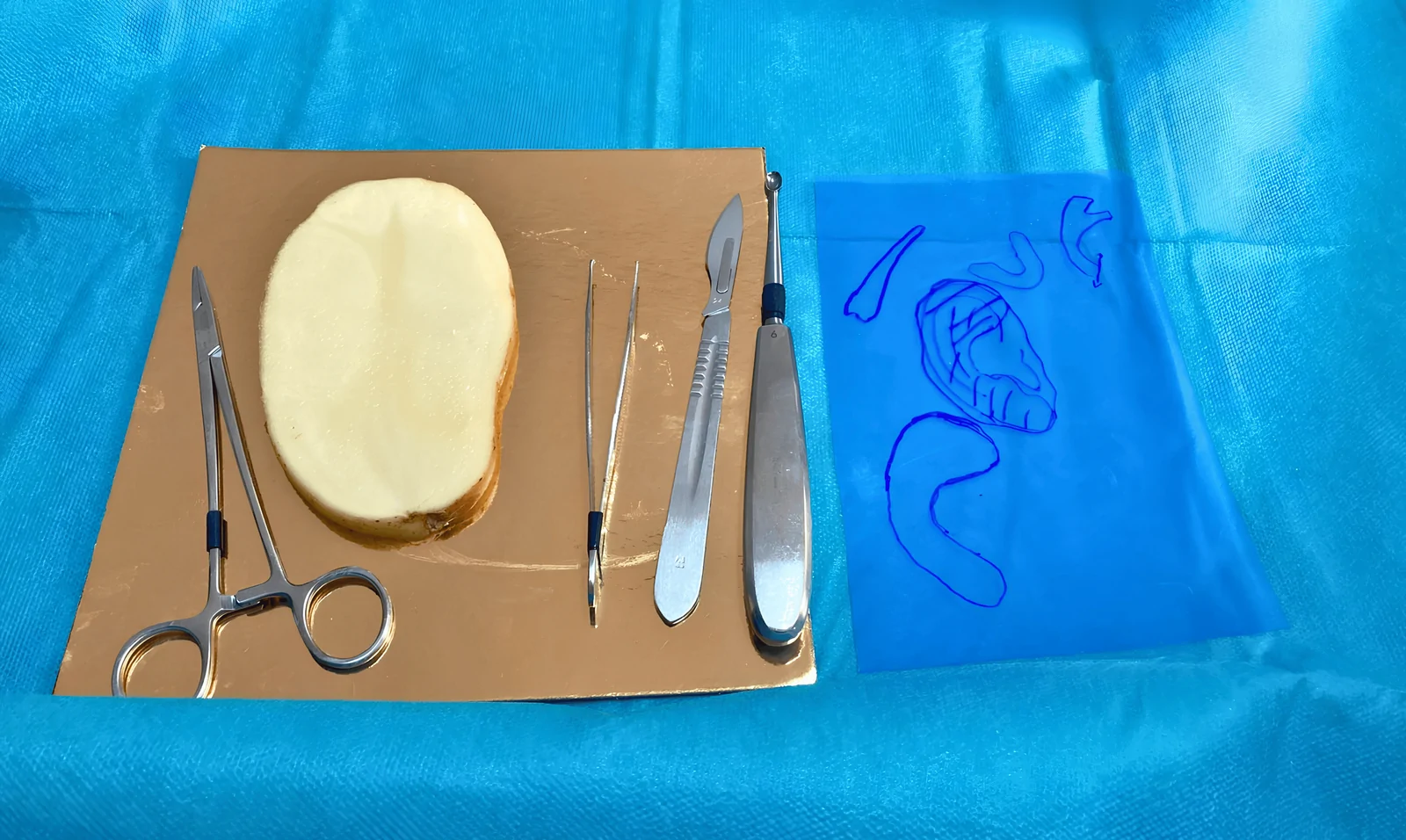
Preparation of low-cost, low-fidelity simulation materials, including a raw potato half-tuber, scalpel blades, gouges, and suturing materials

The complex surgical task was deconstructed into five distinct components for the novice residents: the Base (Socket), the Helix (Rim), the Antihelix (Y-shape), the Tragus-Antitragus complex, and the Projection Block (Shim) (Table 1). The learners first transferred a two-dimensional (2D) paper template of a healthy contralateral ear onto the raw vegetal surface using a transparent film and a surgical skin marker, as demonstrated in Figure 2. They then proceeded with the fine three-dimensional carving of each anatomical relief using the scalpel and bone gouges before completing the final assembly using a stainless-steel wire “frame suture” technique.

**Table 1:** The anatomical correspondence between each framework component, its costal origin in real otopoiesis, its vegetal transposition on the potato model, and the associated carving task

Framework component	Costal origin (surgical)	Anatomical target (vegetal transposition)	Carving task and challenges
Base (socket)	Ribs 6 and 7 (synchondrosis)	Raw potato half-tuber	3D excavation with gouges without base perforation
Helix (rim)	8^th^ rib	Outer peripheral rim and tail	Peripheral thinning with scalpel; high risk of fracture
Antihelix (Y)	Relief cartilage	Main fold and triangular fossa	Sculpting the bifurcation of the antihelix branches
Tragus-antitragus	9^th^ rib	Anterior shield projection	Volumetric adjustment and anterior relief integrity
Projection shim	9^th^ rib	Posterior thickness block	Fixation via "frame suture" to stabilize projection

**Figure 2:**
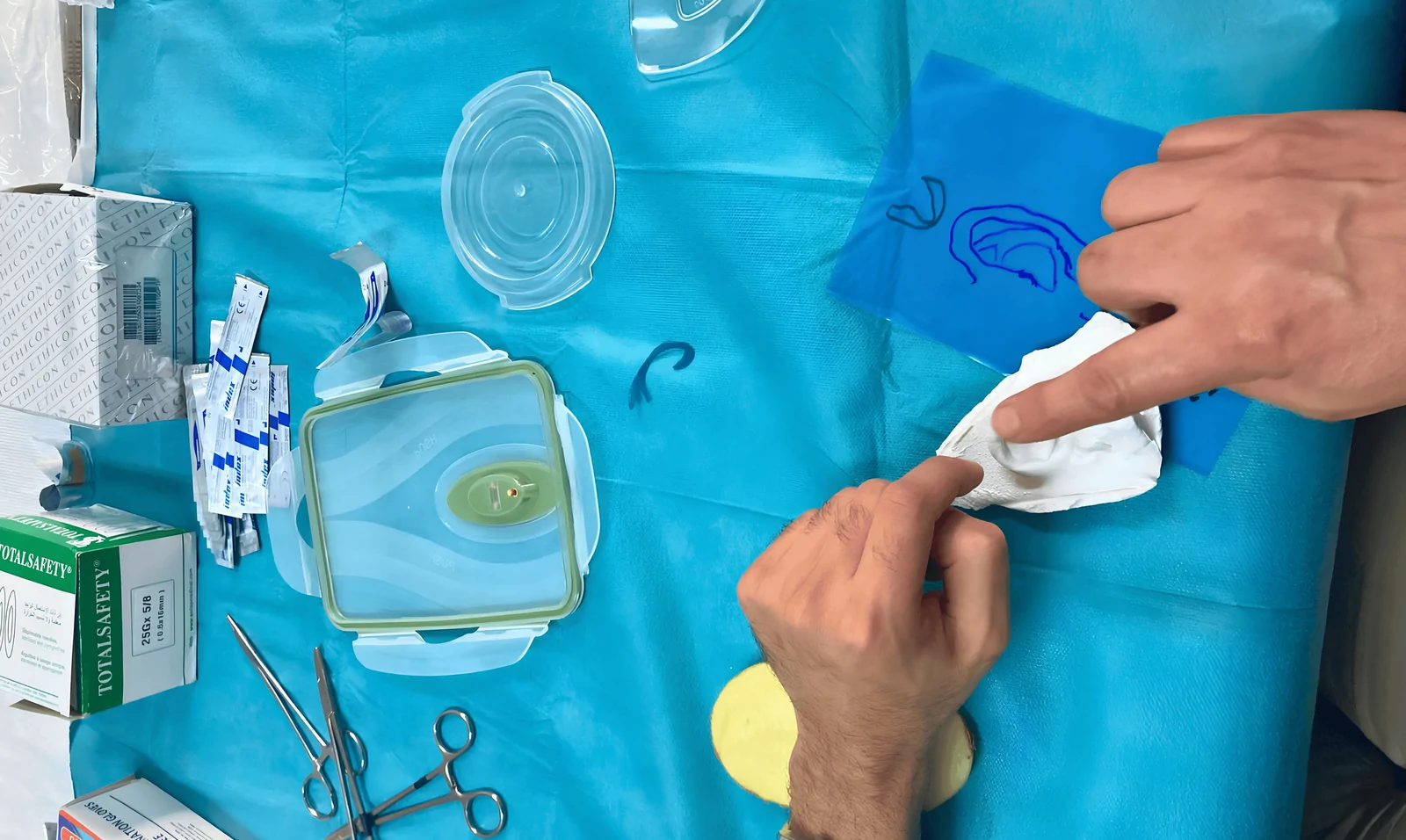
Templating step: transfer of the 2D contralateral ear outline (helix, antihelix, and tragus landmarks) onto the potato surface using a transparent film and surgical skin marker before carving

Evaluation metrics and outcomes

Educational outcomes were assessed using three complementary instruments, each independently verifiable from raw response-level data. First, a 10-item multiple-choice knowledge questionnaire covering auricular embryology, anthropometric norms, standard otoplasty techniques, and the Firmin/Nagata otopoiesis staging sequence was administered anonymously via Google Forms immediately before the theoretical prebriefing (pretest) and again at the conclusion of the workshop (post-test). Fifteen of 16 participants (93.8%) completed the pretest, and eight of 16 (50.0%) completed the post-test; responses were anonymous and could not be matched across the two time points. Second, hands-on skill development was supported through continuous, real-time formative coaching by two senior maxillofacial surgeons circulating among the dyads, correcting technique and verbally validating each completed component. This is reported narratively rather than as a numerical psychometric score, as no calibrated grading instrument was applied. Third, perceived pedagogical utility was documented through an anonymous 11-item Likert-type post-workshop satisfaction survey; nine of 16 participants (56.3%) responded. Descriptive statistics were performed using Microsoft Excel (Microsoft Corp., Redmond, WA, USA).

Cognitive knowledge gains

The mean knowledge score improved from 56.0% (pretest, N=15) to 80.0% (post-test, N=8) across the 10 graded items, with every item improving or remaining stable (Table 2, Figure 3).

**Table 2:** Pretest and post-test knowledge questionnaire results. Percentage of correct responses for each item before (pretest, N=15) and after (post-test, N=8) the workshop, with the correct answer indicated for each of the 10 questions

Question (theme)	Correct answer	Pretest (N=15)	Post-test (N=8)
Cephaloconchal angle (mean value)	C (80°-90°)	13.3% (2/15)	50.0% (4/8)
Number of hillocks of His	E (6)	20.0% (3/15)	87.5% (7/8)
Non-cause of prominent ear	E (tragus agenesis)	66.7% (10/15)	100% (8/8)
Stenström technique principle	C (chondrotomies)	46.7% (7/15)	62.5% (5/8)
Target anomaly of Furnas technique	D (conchal hypertrophy+angle)	53.3% (8/15)	75.0% (6/8)
Most feared postoperative complication	B (perichondritis)	53.3% (8/15)	75.0% (6/8)
Lobular microtia (Nagata)	B	73.3% (11/15)	87.5% (7/8)
Operative stages (Nagata)	B (2 stages)	73.3% (11/15)	75.0% (6/8)
Origin of harvested costal cartilage	C (6^th^-9^th^ ribs)	93.3% (14/15)	100% (8/8)
Interval between the two stages	D (4 months)	66.7% (10/15)	87.5% (7/8)

**Figure 3:**
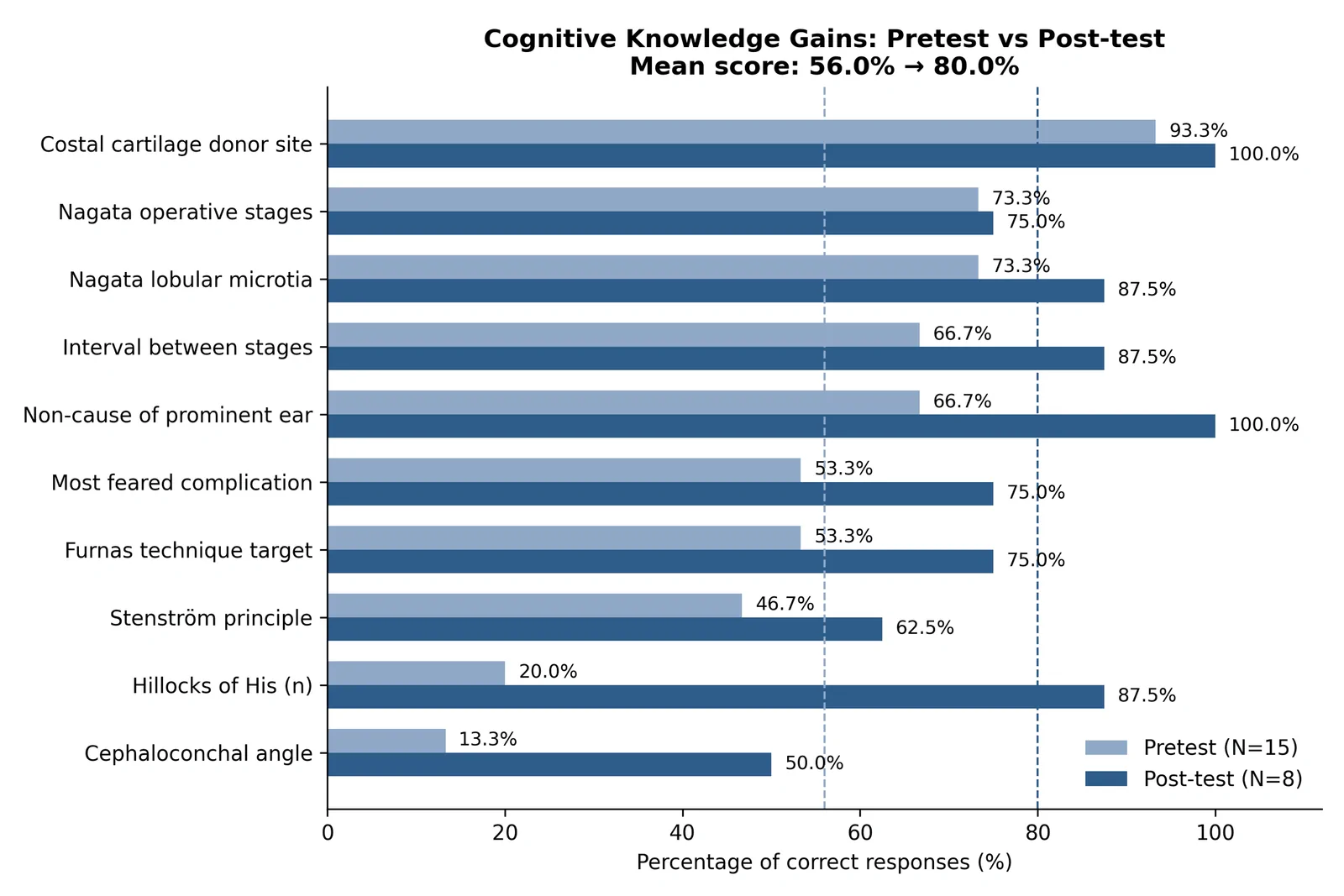
Cognitive knowledge gains between pretest (N=15) and post-test (N=8) across the 10 questionnaire items, sorted by pretest performance. Dashed lines indicate the mean score at each time point (56.0% and 80.0%, respectively)

The largest gains were observed for embryological (hillocks of His: 20.0% → 87.5%) and anthropometric (cephaloconchal angle: 13.3% → 50.0%) items covered explicitly in the prebriefing; items with high baseline familiarity (costal cartilage donor site: 93.3% → 100%) showed a ceiling effect.

Formative technical supervision

Throughout the session, supervising surgeons observed that peripheral thinning of the helix was the most technically demanding step, as the potato’s rigidity and lack of elasticity, in contrast with costal cartilage, made this region prone to fracture under excessive scalpel pressure. This was used as an instructive, low-stakes error signal rather than a scored failure, since residents could restart the affected component at negligible material cost. Base excavation depth was the most common point requiring guidance for junior residents, while senior residents, despite prior surgical experience, encountered the same conceptual difficulty in visualizing the five-component spatial deconstruction of the framework, underscoring that this training addresses a genuine curricular gap independent of seniority.

Satisfaction survey

Analysis of the anonymous satisfaction survey (N=9) showed unanimous agreement on the workshop's spatial and cognitive value: 100% (9/9) agreed the hands-on model improved their 3D spatial understanding of the auricle, and 100% (9/9) reported acquiring the conceptual skills to differentiate simple otoplasty from complete autologous reconstruction. In addition, 77.8% (7/9) reported a clear improvement in theoretical knowledge, consistent with the objective pretest/post-test gain (Figure 4).

**Figure 4:**
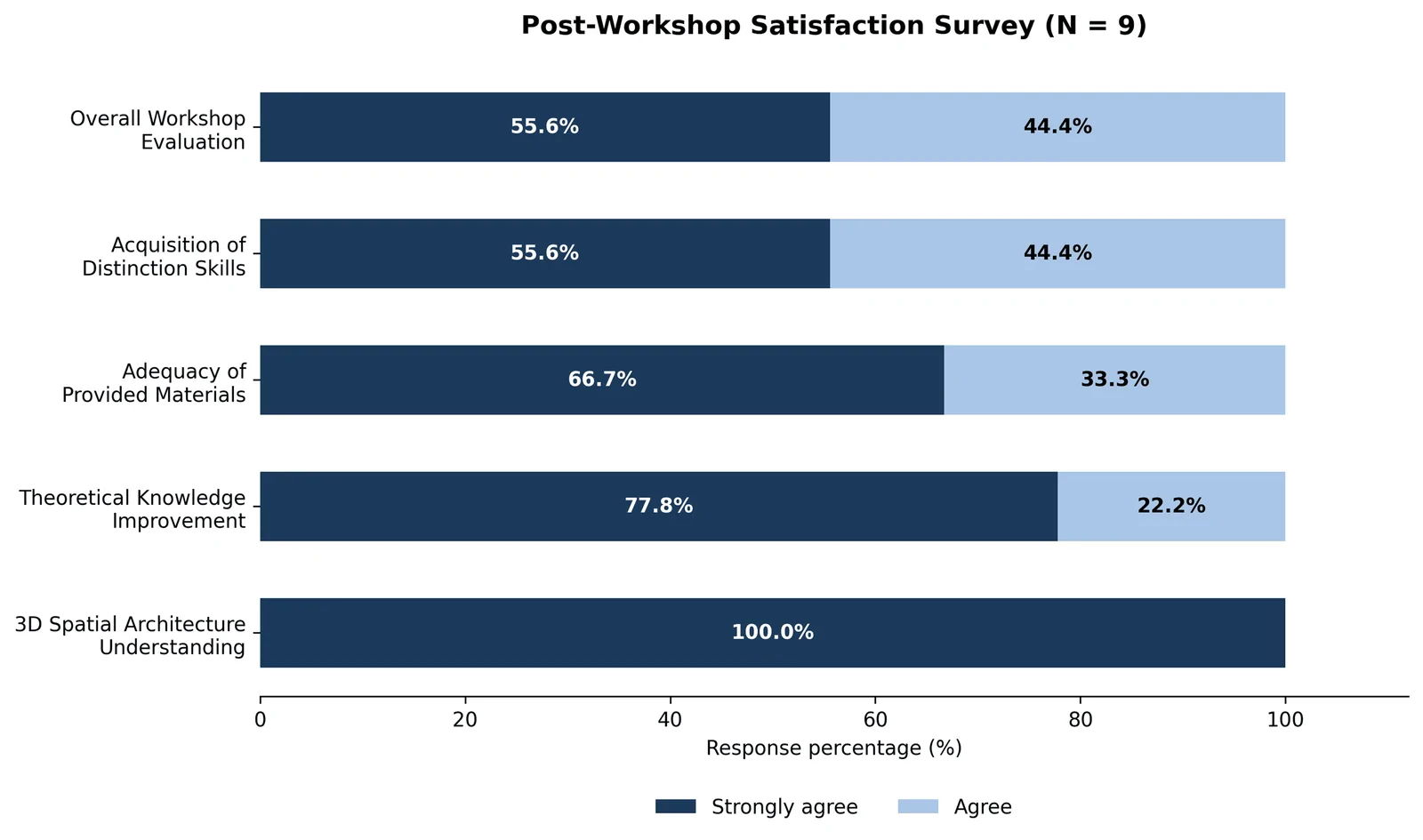
Post-workshop satisfaction survey results (N=9), showing the proportions of “strongly agree” and “agree” responses across five evaluated dimensions

## Discussion

In modern surgical education literature, high-fidelity synthetic models or cadaveric specimens are widely praised for their realistic haptic properties and tactile feedback [8,9], although cadaveric cartilage itself is stiff and brittle and does not fully replicate the handling properties of living donor cartilage. However, the primary pedagogical hurdle for early-stage residents in otopoiesis is not necessarily tissue sensation but rather the heavy cognitive load induced by the complex visuospatial arrangement of the cartilaginous reliefs [4]. The raw potato simulation workshop appears to replicate the essential cognitive steps of Firmin’s anatomical deconstruction [10] at low material cost. Through active, embodied learning, residents physically assimilate the concept that the reconstructed auricular framework is not carved from a single block but assembled by combining five distinct components.

While the material costs of the potato model itself are negligible, the workshop relied on reusable surgical instruments (scalpels, gouges, and sutures) already available within the department; the term “low-cost” is therefore used in preference to “zero-cost” to reflect this. Other easily accessible vegetal materials (e.g., carrot, turnip) could similarly be explored depending on local availability and desired firmness. Animal-derived alternatives, such as porcine costal cartilage or bovine scapular cartilage, have also been used in auricular framework training and may offer haptic properties closer to human costal cartilage, at higher cost and with additional biosafety handling requirements; a direct comparison between vegetal and animal-tissue models would help clarify their relative pedagogical value.

While this educational intervention demonstrates clear feasibility and high user satisfaction, it is critical to moderate claims regarding formal educational effectiveness and construct validity. Although the raw potato offers sufficient firmness to resist scalpel and gouge abrasion, forcing the resident to regulate applied pressure, it lacks the specific anisotropy and elasticity of human rib cartilage. Therefore, this model cannot be claimed to possess complete construct validity without rigorous, direct comparative data. Instead, it serves as an accessible, low-cost baseline training matrix that allows for high-volume, repetitive practice. Qualitative feedback from participants and supervisors suggested that using a common, low-cost vegetal material reduced perceived performance anxiety relative to higher-stakes training contexts, creating an environment in which carving errors could be used as a constructive basis for debriefing rather than being penalized.

Study limitations

Several limitations should be acknowledged. The sample is small (16 residents) and drawn from a single institution, limiting generalizability. Pretest and post-test responses were collected anonymously and could not be matched at the individual level, so the reported knowledge gain reflects an unmatched group-level shift rather than paired individual learning trajectories. Post-test completion (50.0%) was also lower than pretest completion (93.8%), and residents who remained until the end of the session may not have been fully representative of the full cohort. The satisfaction survey response rate (56.3%, 9/16) introduces potential nonresponse bias, as more satisfied participants may have been more likely to respond. Technical skill development was supported through real-time formative supervision rather than a calibrated, standardized assessment instrument; no quantitative measure of carving proficiency is reported, and future iterations should consider a structured, piloted assessment tool if such a measure is required. Finally, the absence of a comparison group trained on higher-fidelity or biological models precludes any conclusion about the transfer of skills acquired using the vegetal model to real surgical performance; prospective, controlled, multi-institutional studies are needed to establish this transfer.

## Conclusions

This educational intervention suggests that low-fidelity vegetal simulation using a raw potato model is feasible, well accepted, and low-cost for introducing early-stage otopoiesis concepts. While it cannot replace the haptic fidelity of real cartilage or higher-fidelity synthetic models, it appears to help bridge the gap between theoretical anatomy and basic procedural understanding in an accessible and low-pressure learning format. Further controlled, multi-institutional research is needed to determine whether the conceptual and technical gains observed here translate into improved performance on higher-fidelity models or in clinical practice.
